# The Chromosomal Passenger Complex Activates Polo Kinase at Centromeres

**DOI:** 10.1371/journal.pbio.1001250

**Published:** 2012-01-24

**Authors:** Mar Carmena, Xavier Pinson, Melpi Platani, Zeina Salloum, Zhenjie Xu, Anthony Clark, Fiona MacIsaac, Hiromi Ogawa, Ulrike Eggert, David M. Glover, Vincent Archambault, William C. Earnshaw

**Affiliations:** 1 Wellcome Trust Centre for Cell Biology, University of Edinburgh, Edinburgh, Scotland, United Kingdom; 2 Institut de Recherche en Immunologie et en Cancérologie, Université de Montréal, Montréal, Québec, Canada; 3 Dana-Farber Cancer Institute and Department of Biological Chemistry and Molecular Pharmacology, Harvard Medical School, Boston, Massachusetts, United States; 4 Cancer Research UK, Cell Cycle Genetics Research Group, Department of Genetics, University of Cambridge, Cambridge, United Kingdom; 5 Département de Biochimie, Université de Montréal, Montréal, Québec, Canada; Stowers Institute for Medical Research, United States of America

## Abstract

The coordinated activities at centromeres of two key cell cycle kinases, Polo and Aurora B, are critical for ensuring that the two sister kinetochores of each chromosome are attached to microtubules from opposite spindle poles prior to chromosome segregation at anaphase. Initial attachments of chromosomes to the spindle involve random interactions between kinetochores and dynamic microtubules, and errors occur frequently during early stages of the process. The balance between microtubule binding and error correction (e.g., release of bound microtubules) requires the activities of Polo and Aurora B kinases, with Polo promoting stable attachments and Aurora B promoting detachment. Our study concerns the coordination of the activities of these two kinases in vivo. We show that INCENP, a key scaffolding subunit of the chromosomal passenger complex (CPC), which consists of Aurora B kinase, INCENP, Survivin, and Borealin/Dasra B, also interacts with Polo kinase in Drosophila cells. It was known that Aurora A/Bora activates Polo at centrosomes during late G2. However, the kinase that activates Polo on chromosomes for its critical functions at kinetochores was not known. We show here that Aurora B kinase phosphorylates Polo on its activation loop at the centromere in early mitosis. This phosphorylation requires both INCENP and Aurora B activity (but not Aurora A activity) and is critical for Polo function at kinetochores. Our results demonstrate clearly that Polo kinase is regulated differently at centrosomes and centromeres and suggest that INCENP acts as a platform for kinase crosstalk at the centromere. This crosstalk may enable Polo and Aurora B to achieve a balance wherein microtubule mis-attachments are corrected, but proper attachments are stabilized allowing proper chromosome segregation.

## Abstract 2

INCENP acts as a protein scaffold that integrates the functions of two crucial mitotic kinases, Aurora B and Polo, at centromeres of mitotic chromosomes.

## Author Summary

When cells divide, their chromosomes segregate to the two daughter cells on the mitotic spindle, a dynamic macromolecular scaffold composed of microtubules. Each chromosome consists of two sister chromatids. Microtubules attach to the chromatids at structures called kinetochores, which assemble at the surface of the constricted centromere region where the sister chromatids are most closely paired. To segregate correctly, sister kinetochores must attach to microtubules emanating from opposite spindle poles. Kinetochore attachment to microtubules occurs randomly and mistakes occur frequently. For example, both sister kinetochores may attach to one pole, or one kinetochore may attach to both poles simultaneously. Two protein kinases, Aurora B and Polo, have essential roles in regulating this process: Aurora B triggers the release of incorrect attachments and Polo strengthens the grip that correctly attached kinetochores have on microtubules. In this work, we have investigated the potential functional links between these two crucial enzymes at centromeres in cells of the fruitfly. We found that early in division, Aurora B and Polo both interact with a structural partner protein named INCENP at centromeres. This allows Aurora B to phosphorylate Polo, thereby activating it. We show that coordinating the activities of these two central mitotic kinases is crucial for successful cell division, and that this mechanism is conserved in human cells.

## Introduction

Executive decisions concerning when cells enter and exit mitosis are made by Cdk1 with cyclins A and B as cofactors. Once cells have entered mitosis, Plk1 and the Aurora kinases direct spindle formation, regulate chromosome attachments to spindle microtubules, ensure the operation of the spindle checkpoint, and enable daughter cells to complete cytokinesis (reviewed in [1]–[4]). Plk1 and Aurora A also function in the regulation of mitotic entry (reviewed in [5]).

In higher eukaryotes, Plk1 and Aurora B have potentially antagonistic activities during the early stages of chromosome attachment and alignment on the mitotic spindle. Plk1 phosphorylation of kinetochore components and microtubule plus-end-associated proteins is required for the establishment of stable kinetochore-microtubule (KT-MT) interactions. Electron micrographs of human cells treated with the Plk1 inhibitor BI2536 show fewer microtubule connections per kinetochore [6]. Tension-sensitive phosphorylation of BubR1 by Plk1 regulates the initial stability of KT-MT interactions [7], as do phosphorylation of CLIP-170 [8] and NudC [9]. Plk1 also phosphorylates components of the Ska and KNL-1/Mis12/Ndc80 (KMN) kinetochore complexes as well as centromere proteins CENP-B, CENP-C, CENP-E, and CENP-F. However, the function of these phosphorylations is not known [10].

The chromosomal passenger complex (CPC), consisting of Aurora B kinase, INCENP, Survivin, and Borealin [11], has a role in the correction of kinetochore-microtubule attachment errors by promoting the release of kinetochore-microtubule attachments [11]–[15]. The localization of the CPC relative to kinetochores is critical for regulation of kinetochore-microtubule attachments [16]. CPC targeting to inner centromeres occurs as a result of Survivin binding to histone H3 phosphorylated on Thr3 by Haspin kinase [17]–[19] and is helped by an Aurora B-dependent positive feedback loop [20].

The temporal and spatial regulation of Plk1 activation is complex. While Polo activity is not required for mitotic entry in unperturbed cell cycles, its activation by T-loop phosphorylation is needed for its functions during mitosis. At centrosomes, Aurora A kinase-Bora phosphorylates Plk1 on Thr210, thereby activating Plk1 at the G2-M transition in human cells [21],[22]. Subsequently, Plk1 triggers the degradation of both Bora [23],[24] and Aurora A [25]. The regulation of Plk1 activity at kinetochores is a critical and largely unstudied question.

Here, we have examined the mechanism of Polo kinase activation at Drosophila and human kinetochores. We show that Aurora B is required for Polo T-loop phosphorylation at the centromere, but not at centrosomes. Our studies identify a new regulatory link between the Aurora B and Polo kinases mediated by INCENP. Furthermore, we demonstrate that this mechanism of regulation of Polo kinase at the kinetochore by the CPC is conserved in human cells. These results support our previous hypothesis that INCENP acts as a platform coordinating the activities of these kinases on chromosomes during early mitosis [26].

## Results

### Polo Kinase Localizes to the Centromere/Kinetochore Region Before the CPC Does

**Figure 1:**
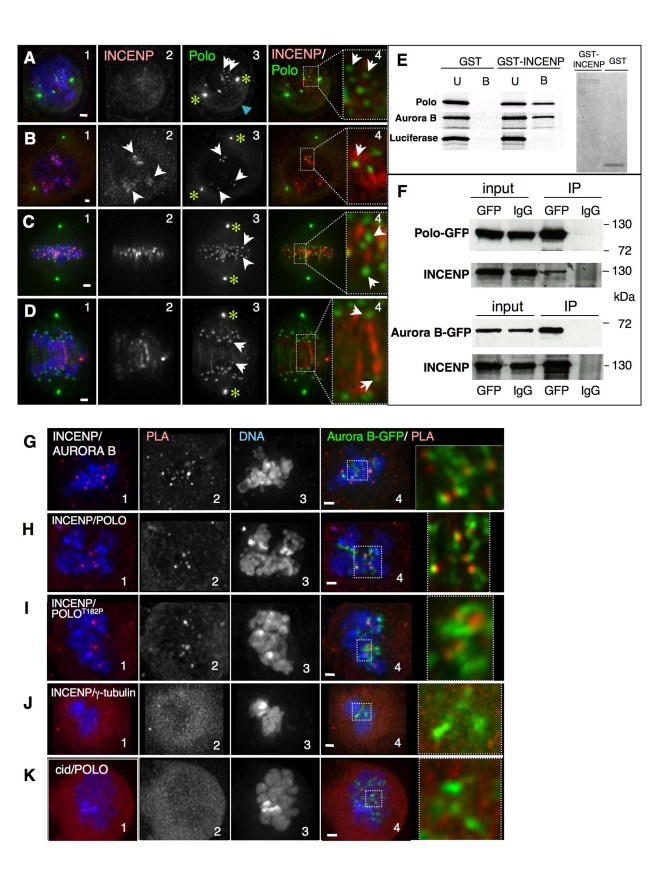
INCENP colocalizes with Polo in early mitosis and interacts with Polo at the inner centromere. (A) Early prophase, Polo is localized at centrosomes (asterisks), nuclear envelope (blue arrowhead), and starts appearing on chromosomes (white arrows), while INCENP is not yet clearly associated with chromatin. (B) Late prophase, INCENP appears associated with heterochromatin but not yet concentrated at the inner centromere (2, arrows), while Polo is already concentrated in dots colocalizing with INCENP-positive regions (3, white arrows). (C) Metaphase, INCENP at the inner centromere region stretching between Polo-positive kinetochores (arrows). (D) Early anaphase, INCENP decorates thread-like structures joining the segregating Polo-positive sister kinetochores (arrows). Green, Polo-GFP; red, INCENP. Scale bar = 1 µm. (E) In vitro pulldown assays. GST or GST-tagged INCENP were incubated with in vitro translated ^35^S labelled Polo, Aurora B, or Luciferase. U, unbound fraction; B, bound fraction. Right panel, Coomasie stained gel showing the proteins used for the pulldown. (F) Immunoprecipitation assays. Protein extracts from Polo-GFP (upper panel) or Aurora B-GFP (lower panel) stably transfected cell lines were used in IP experiments using anti-GFP or IgG (Input, whole cell extract, 1% of total loaded; IP, bound fraction, 20% of total loaded). (G–K) Proximity ligation assay (PLA) using antibodies against (G) INCENP and Aurora B (positive control); (H) INCENP and Polo; (I) INCENP and Polo^T182Ph^; (J) INCENP and γTubulin (negative control); and (K) Polo and CID (Negative control). Zoomed panels show colocalization of the PLA signal (red) with Aurora B-GFP. Scale bar = 1 µm.

As a starting point to examine the relationship between Polo and the CPC in Drosophila, we compared their localization in space and time. INCENP and the CPC are diffuse during early prophase in Drosophila cultured cells (Figure 1A2). In contrast, Polo kinase in these early stages is localized at centromeres (Figure 1, arrows; Figure S1A), at centrosomes, where it colocalizes with Aurora A (Figure 1A–D, asterisks; Figure S2), and also at the nuclear envelope (Figure 1A3, blue arrowhead), where it has been proposed to promote nuclear envelope breakdown (NEB) [8].

Later during prophase and early in prometaphase, INCENP concentrates at specific, brightly stained chromosomal regions that probably correspond to heterochromatin. At this time, Polo kinase is already concentrated at centromeres (Figure 1B3). This is the earliest stage at which we observe partial colocalization between Polo (Figure 1B3,4 white arrows) and the CPC.

In metaphase, when chromosomes are bioriented and under tension, INCENP is concentrated on inner centromeric threads that extend between bioriented sister kinetochores, running parallel to microtubules (Figure 1C2,4; Figure S1D). At this stage, most chromosome-associated Polo is detected in the outer kinetochore (Figure 1C3,4, arrows) and does not colocalize with INCENP. Later, in early anaphase, INCENP and Polo are observed on threads parallel to central spindle microtubules (Figure 1D and unpublished data), although prominent Polo labelling is still detected at the kinetochore.

We conclude that Polo concentrates at centromeres before INCENP does and that both proteins transiently colocalize there during early prometaphase.

### INCENP and Polo Interact in vitro and in vivo at the Centromere in Early Mitosis

To ask if this colocalisation of INCENP and Polo reflects a direct interaction between the two proteins, soluble, bacterially expressed, full-length Drosophila GST-INCENP (Figure 1E right panel) was mixed with in vitro-translated Polo kinase using Aurora B and luciferase as positive and negative controls, respectively. The mixture was then incubated with glutathione beads and bound proteins detected by SDS-PAGE. Robust binding was observed between GST-INCENP and Polo or Aurora B, but not with the luciferase control (Figure 1E). Interestingly, this interaction did not require CDK phosphorylation of INCENP, as previously described for the binding of mammalian INCENP to Plk1 [27].

We confirmed that a physical interaction between INCENP and Polo also occurs in vivo by immunoprecipitation from cell extracts. Cell lines stably expressing either Polo-GFP or Aurora B-GFP (positive control) were lysed and the tagged protein immunoprecipitated with anti-GFP. INCENP was readily detected in both immunoprecipitates by immunoblotting (Figure 1F).

To determine more precisely when and where INCENP and Polo interact during the cell cycle, we used a proximity ligation assay (PLA) to map sites where INCENP, Polo, and Aurora B are in close proximity. PLA is based on conventional double staining using primary antibodies raised in different species. The secondary antibodies used for detection are tagged with short DNA oligonucleotides. If those oligonucleotides are close enough to allow them to be bridged by hybridization with circle-forming oligonucleotides (distance between antigens of 10–30 nm, [28]), the circle can be amplified by rolling circle DNA synthesis and a positive PLA signal is obtained. That signal requires not only the close proximity of the antigens but also their favourable spatial conformation and absence of structural obstacles so that the oligos can interact and subsequent reactions take place. Thus, only a subset of actual interactions between proteins is detected with the PLA technique.

We validated the PLA assay by first confirming the known interaction between endogenous INCENP and Aurora B [29]–[32]. Indeed, we readily observed PLA signals associated with chromosomes in prophase and prometaphase cells (Figure 1G1). This confirmed a recent study that found positive PLA signals between various members of the CPC in all phases of mitosis in human cells [33].

In Drosophila, the positive PLA signals overlapped with Aurora B-GFP, confirming that epitopes on INCENP and Aurora B are in close proximity in the inner centromere (Figure 1G4). A parallel assay using antibodies against INCENP and GFP confirmed this close association of INCENP with exogenous GFP-tagged Aurora B (unpublished data). In two negative controls, we failed to observe PLA signals using antibodies to INCENP and γ-tubulin and between Polo and the centromere histone CENP-A/CID (Figure 1J,K).

We next used PLA to define where in cells interactions occur between INCENP and Polo. Cells co-stained for INCENP and Polo showed PLA signals on chromosomes in early mitosis (Figure 1H1). These signals colocalized with Aurora B-GFP, confirming that the interaction occurs at inner centromeres (Figure 1H4). PLA also detected a close association between INCENP and Polo^T182ph^, the activated form of Polo kinase, using an anti-phospho-epitope-specific antibody (Figure 1I1, see validation of the antibody below). These positive PLA signals were also present in inner centromeres (Figures 1I4).

We conclude that INCENP and Polo physically interact and are in close proximity at inner centromeres during early mitosis in Drosophila.

### INCENP Is Required for Polo Kinase Activation at the Inner Centromere

**Figure 2:**
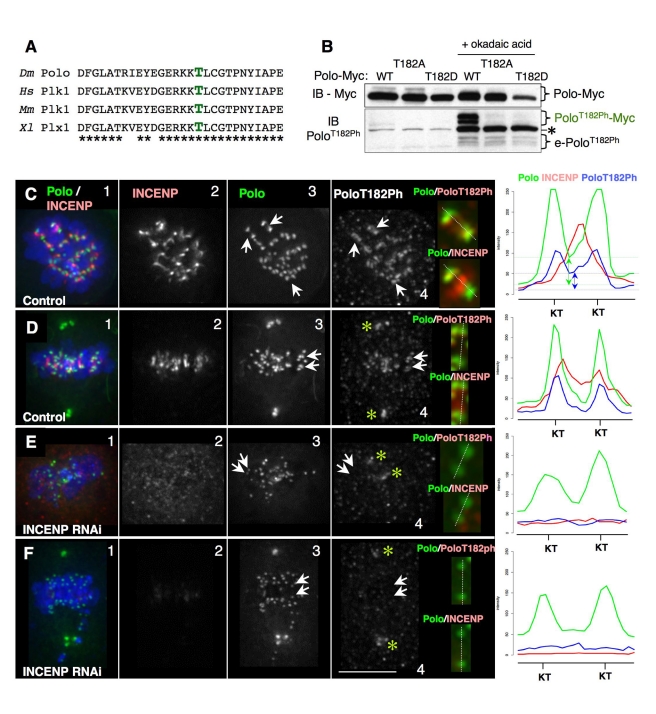
INCENP is required for the activation of Polo kinase at the inner centromere. (A) Sequence alignment showing the conservation of Thr182 across species. (B) Immunoblot of cell lines stably expressing wild-type, T182A, or T182D Polo in the absence or presence of okadaic acid. The phosphoespecific antibody anti-Plk1^T210Ph^ recognises *Drosophila* Polo^T182Ph^, both Myc-tagged and endogenous (e). Both endogenous and Myc-tagged Polo^T182Ph^ were detected as a doublet, suggesting that they can be modified at another site. The asterisk indicates a non-specific band that does not disappear after Polo RNAi. This band increases following okadaic treatment, and therefore could correspond to a non-specific phospho-epitope. (C–F) Control or RNAi-treated DMel-2 cells stably expressing Polo-GFP showing colocalization of INCENP and Polo/Polo^T182Ph^. Arrows point to chromosomes shown in zoomed images. Linescans show fluorescence intensity across the kinetochores (dashed lines). (C) Control prometaphase. Polo^T182Ph^ is visibly enriched at the inner centromere compared to Polo (arrows). Linescans show both Polo and Polo^T182Ph^ are present at the inner centromere (double-ended arrows show difference in intensity with respect to background levels: green, Polo blue, Polo^T182Ph^). (D) Control metaphase. Asterisks point to centrosomes, and Polo^T182Ph^ is virtually undetectable in the inner centromere (linescan; note that intensity drops to background level). (E,F) INCENP RNAi-treated cells. Asterisks point to Polo^T182Ph^ on centrosomes (note absence from kinetochores, also shown in linescans). Zoomed images show localization at the centromere/kinetochore of the indicated proteins.

The association between INCENP and Polo described above suggested that INCENP and other CPC components might have a role in Polo activation by T-loop phosphorylation. To test this hypothesis, we asked whether reducing INCENP protein levels by RNAi affected the localization and activation of Polo kinase, as detected by monitoring Polo T-loop phosphorylation. Plk1 phosphorylation on the highly conserved T-loop residue Thr-210 (Thr-182 in Drosophila Polo, Figure 2A) is crucial for kinase activation [34]. T182 of Drosophila Polo is a major phosphorylation site detected by mass spectrometry, and a phosphomimetic mutation at that site (Polo^T182D^) increases Polo kinase activity in vitro (VA, unpublished observations).

To examine Polo activation, we used a phospho-epitope-specific antibody raised against human Plk1^T210ph^ that also recognises Drosophila Polo^T182ph^ (Figure 2B). In asynchronous cultures, Polo^T182ph^ was barely detectable by Western blotting. However, we could readily observe endogenous Polo^T182ph^ using this antibody after treatment with the phosphatase inhibitor okadaic acid (OA; Figure 2B). This signal disappeared following Polo depletion by RNAi, confirming that it comes from the Polo protein (see below). Moreover, Polo-Myc gives a signal at the expected higher molecular weight, while Polo^T182A^-Myc and Polo^T182D^-Myc are not recognized by this antibody (Figure 2B). In these experiments, Polo was detected as a doublet by Western blotting. This mobility shift is not caused by T182 phosphorylation and may be caused by an as-yet uncharacterized modification.

We used this antibody to examine the distribution of Polo^T182ph^ by immunofluorescence in cycling DMel-2 cells that had not been treated with okadaic acid. The antibody detected Polo^T182ph^ at centromeres/kinetochores, centrosomes, the cleavage furrow, and midbody. This staining was largely abolished following Polo RNAi-depletion (Figure S3).

Although most Polo accumulates at prometaphase kinetochores (Figure 2C3; Figure S2B,C; Figure S4A–C linescans), a minor fraction of the kinase localizes to inner centromeres (Figure 2C3 arrows; Figure 2C linescan). Indeed, the active kinase (detected with anti-Polo^T182ph^) is clearly detectable at inner centromeres (Figure 2C4+inset, arrows), where it colocalizes with INCENP, as predicted by the PLA results (Figure 2C4; linescan). We first detect this inner-centromeric pool of active Polo in late prophase cells (Figure S4A–C). Polo^T182ph^ is no longer detected at the inner centromeres of chromosomes aligned at the metaphase plate. Instead it accumulates at kinetochores in metaphase cells (Figure 2D4, arrows; linescan).

Depletion of INCENP by RNAi substantially reduced levels of activated Polo ^T182ph^ at kinetochores (Figure 2E4,F4; linescans). In contrast, total Polo localized normally to kinetochores following INCENP knockdown (Figure 2E3,F3; linescans). This is consistent with the observation that Polo localization to this region precedes that of INCENP (Figure 1A). Importantly, we could still readily detect active Polo^T182ph^ at centrosomes of cells following INCENP knockdown (Figure 2E4,F4, asterisks).

These experiments reveal that INCENP is required for T182 phosphorylation and activation of Polo kinase at inner centromeres in early mitosis. Activation of centrosomal Polo does not require INCENP.

### Polo T-Loop Phosphorylation Is Required for Mitotic Progression But Not For Mitotic Entry

**Figure 3:**
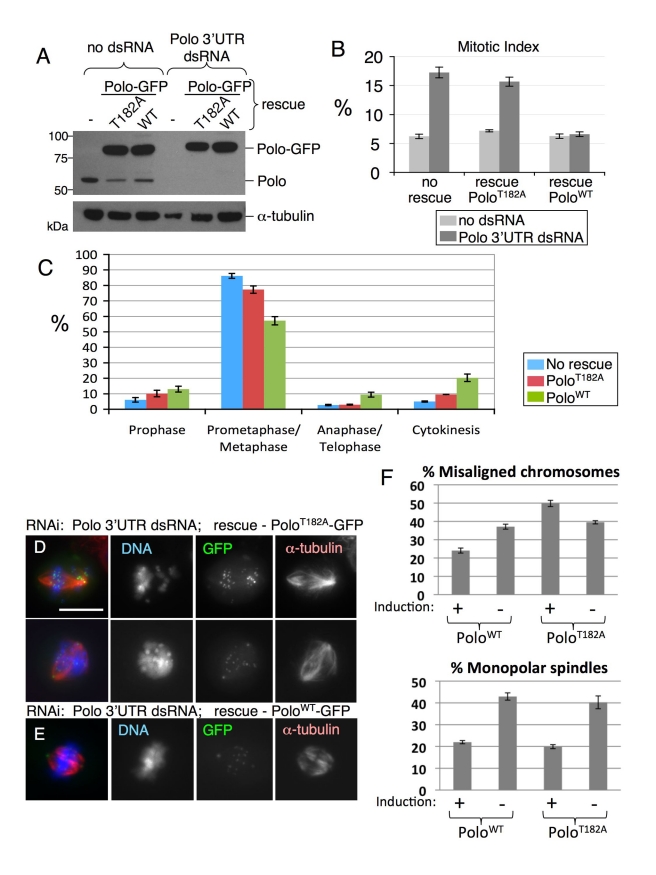
Polo T-loop phosphorylation is required for mitotic progression but not for mitotic entry. (A) Stable cell lines allowing the copper-inducible expression of Polo^WT^-GFP or Polo^T182A^-GFP (or control DMel-2 cells) were treated with CuSO_4_ for 1 d and transfected with Polo 3′UTR dsRNA the next day in presence of CuSO_4_. Four days later, cells were analyzed by immunoblotting. (B) The same cells were analyzed by immunofluorescence to measure the mitotic index (± S.E.M.) using anti-phospho-Histone H3 staining. Note that the expression of Polo^WT^-GFP, but not Polo^T182A^-GFP, rescued the mitotic index in cells depleted of endogenous Polo. (C) Quantification of the phase distribution of mitotic cells after staining for α-tubulin and DNA (± S.E.M.). Cells expressing Polo^T182A^-GFP accumulate in prometaphase/metaphase compared with Polo^WT^-GFP cells. (D) Cells expressing Polo^T182A^-GFP and depleted of endogenous Polo (D) showed aberrant prometaphase/metaphase figures, with scattered chromosomes, whereas cells expressing Polo^WT^-GFP progressed into a normal metaphase (E). Scale bar = 10 µm. (F) Quantification of defects in chromosome alignment and bipolar spindle formation in the different experimental conditions, where all cells were depleted of endogenous Polo by 3′UTR dsRNA. Error bar = SEM.

In order to investigate the function(s) of Polo T182 phosphorylation in mitosis, we established stable cell lines allowing inducible expression of Polo^WT^-GFP or Polo^T182A^-GFP. Endogenous Polo could be depleted in those cells by RNAi against the 3′UTR of the native transcript (Figure 3A). Expression of Polo^WT^-GFP rescued the viability and proliferation of cells depleted of endogenous Polo. However, expression of Polo^T182A^-GFP did not, and cells died (unpublished data). Thus, Polo T-loop phosphorylation is essential for viability.

Polo-depleted cells accumulated in mitosis, exhibiting phenotypes similar to those observed for the first *polo* mutants [35]. Expression of Polo^WT^-GFP restored mitotic progression in cells depleted of endogenous Polo (Figure 3B), but expression of Polo^T182A^-GFP did not. Cells expressing only Polo^T182A^-GFP accumulated in prometaphase/metaphase (Figure 3C), often with unaligned chromosomes (Figure 3D–F). Interestingly, while the loss of Polo led to an increase in monopolar spindles, substitution of endogenous Polo with Polo^T182A^-GFP did not (Figure 3D–F). This suggests that T-loop phosphorylation of Polo may be dispensable for its role in bipolar spindle assembly.

The observation that INCENP-dependent activation of Polo by phosphorylation at T182 at centromeres/kinetochores is required for chromosome alignment in prometaphase is consistent with the known role of Polo in regulating kinetochore function.

### Aurora B Activity Is Required for Polo Kinase Activation at Centromeres

**Figure 4:**
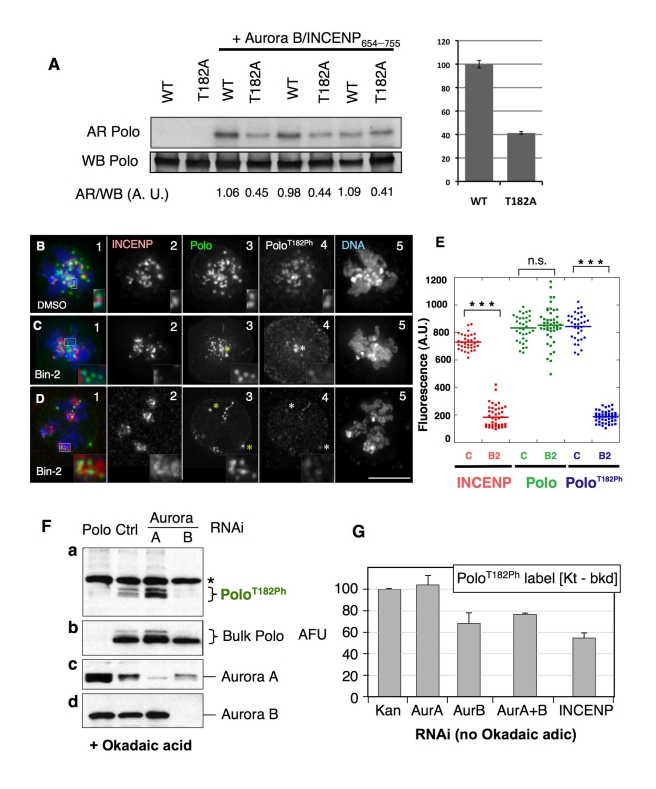
Aurora B activity is required for the activation of Polo kinase at the inner centromere. (A) Aurora B phosphorylates Polo kinase in vitro. Bacterially expressed HIS-Polo or HIS-Polo^T182A^ (which is catalytically inactive and therefore unable to autophosphorylate) were incubated with (or without) Drosophila Aurora B in complex with a fragment of INCENP (residues 654–755) in presence of ^32^P-g-ATP, in triplicate. Reaction products were resolved by SDS-PAGE transferred to nitrocellulose and analyzed by autoradiography (AR) and anti-Polo Western blot (WB). Quantitative measurements of signals were obtained (see Materials and Methods), and the ratios were calculated for each reaction (AR/WB, A.U.: arbitrary units). Right, average values for the relative phosphorylatin of Polo^WT^ and Polo^T182A^ by Aurora B. Error bars, SEM. (B–D) DMel-2 cells stably expressing Polo-GFP treated with (B) DMSO or (C–D) Binucleine-2, immunostained for INCENP, Polo, and Polo^T182Ph^ (insets: zoomed images of kinetochores). In (C–D) asterisks indicate centrosomes. Merged images show INCENP/Polo/DNA. Zoomed images in (C–D) insets show examples of kinetochore pairs showing decreased levels of Polo^T182Ph^. (E) Dot plot showing the quantification of INCENP/Polo/Polo^T182Ph^ signal intensity at the kinetochore (*t* test: *** *p*<0.0001; n.s., not significant; *p* = 0.4028). Signal intensities for individual kinetochores were measured using the SoftWorx Data Inspector tool; average background was subtracted; data was plotted using KaleidaGraph software. (F) RNAi depletion of Aurora B, but not Aurora A, strongly reduces Polo^T182Ph^ levels in DMel-2 cells treated with okadaic acid. Cells were transfected with the indicated dsRNAs for 4 d, and 100 nM okadaic acid added for 4 h before immunoblotting to improve visualization of phosphorylated Polo. A dsRNA against the Kanamycin resistance bacterial gene was used as a negative control. Asterisks: non-specific bands. Both bulk Polo and PoloT^182ph^ appear as doublets. (G) RNAi depletion of Aurora B or INCENP, but not Aurora A, reduces Polo^T182Ph^ levels at centromeres/kinetochores. Cycling cells were treated with the indicated dsRNAs for 3 d (immunoblots are shown in Figure S6B) and Polo^T182Ph^ was detected by immunofluorescence. Levels of Polo^T182Ph^ at centromeres/kinetochores in prometaphase and metaphase cells were measured at individual kinetochores using Image J, subtracting background (Kt-bkd). Asterisks indicate centrosomes. Error bars = S.E.M.

Because the best known role of INCENP is to activate Aurora B kinase in the CPC, we next asked whether Aurora B has a role in Polo T-loop phosphorylation at centromeres. Drosophila Polo T182 (corresponding to human Plk1 T210) is preceded by a conserved stretch of basic residues resembling the consensus site for Aurora kinases (Figure 2A) [31],[36],[37]. Indeed, Drosophila Aurora B complexed with a fragment of INCENP can directly phosphorylate Polo in vitro (Figure 4A). A T182A mutation in the Polo used as a substrate reproducibly reduced its phosphorylation by about one half. Thus, Polo T182 is a major phosphorylation site for Aurora B (Figure 4A). Similar results were obtained using human Aurora B on GST-Polo^WT^ or GST-Polo^T182D^ (unpublished data).

Kinase inhibition studies suggest that Aurora B is responsible for Polo^T182^ phosphorylation in vivo. Binucleine 2 is the only specific Aurora B kinase inhibitor described to date that is effective in Drosophila cells [38],[39]. When DMel-2 cells were treated with 20 µM Binucleine 2 for 2 h, H3^S10ph^ was undetectable in mitotic cells (unpublished data; [39]) and INCENP and Aurora B were dispersed in clumps on the chromosomes ([38],[39]; Figure S5B,C, compare with Figure S5A). Both of these phenotypes are characteristic of the loss of Aurora B function [40],[41].

Aurora B kinase activity is required for Polo activation at kinetochores, and levels of kinetochore-associated Polo^T182ph^ were greatly reduced in Binucleine 2-treated mitotic cells (Figure 4B4–D4; Figure 4E). In contrast, we observed no obvious difference in the localization of bulk Polo kinase in those cells (Figure 4B3–D3; Figure 4E). Importantly, as in the case of INCENP RNAi, we could still detect activated Polo kinase at centrosomes in the same cells (Figure 4B4–D4 asterisks).

As independent confirmation of the inhibitor studies, RNAi-mediated depletion of Aurora B also led to disappearance of the Polo^T182ph^ signal observed in Western blots after OA treatment of cells, while total Polo levels remained unchanged (Figure 4F). In striking contrast, the Polo^T182ph^ signal actually increased after partial Aurora A depletion (Figure 4Fa), perhaps because cells accumulated in mitosis.

The above results suggested that Aurora B rather than Aurora A plays a major role to promote Polo^T182^ phosphorylation at centromeres in Drosophila cells. In order to exclude that our failure to detect Polo^T182ph^ by Western blotting following Aurora B depletion was due to a cell cycle block outside mitosis caused by OA treatment, we examined the effects of RNAi depletion of Aurora A, Aurora B, and INCENP on the Polo^T182ph^ signal at centromeres in individual mitotic cells without okadaic acid treatment. Brief (3 d) dsRNA treatments were used to avoid an accumulation of binucleate cells caused by failure in CPC function in cytokinesis.

Depletion of Aurora B or INCENP led to a significant reduction of the Polo^T182ph^ signal at centromeres (Figure 4G). This effect was specific to centromeres, and Polo^T182ph^ levels at centrosomes were unaffected following depletion of Aurora B or INCENP (Figure S6A). In contrast, Aurora A depletion had no effect on levels of Polo^T182ph^ at centromeres, but led to a modest reduction in Polo^T182ph^ levels at centrosomes.

Together, these results confirm that Aurora B and INCENP are required for Polo activation at the centromere/kinetochore in early mitosis and strongly implicate Aurora B as the kinase responsible.

### The CPC Is Required for Polo Activation at Kinetochores in Larval Neuroblasts

The CPC is required for Polo kinase activation at centromeres in live animals, and not only in aneuploid cultured cells. To demonstrate this, we examined flies homozygous for the hypomorphic female-sterile allele *incenp^QA26^*, a point mutation in the highly conserved IN-box domain [42].

**Figure 5:**
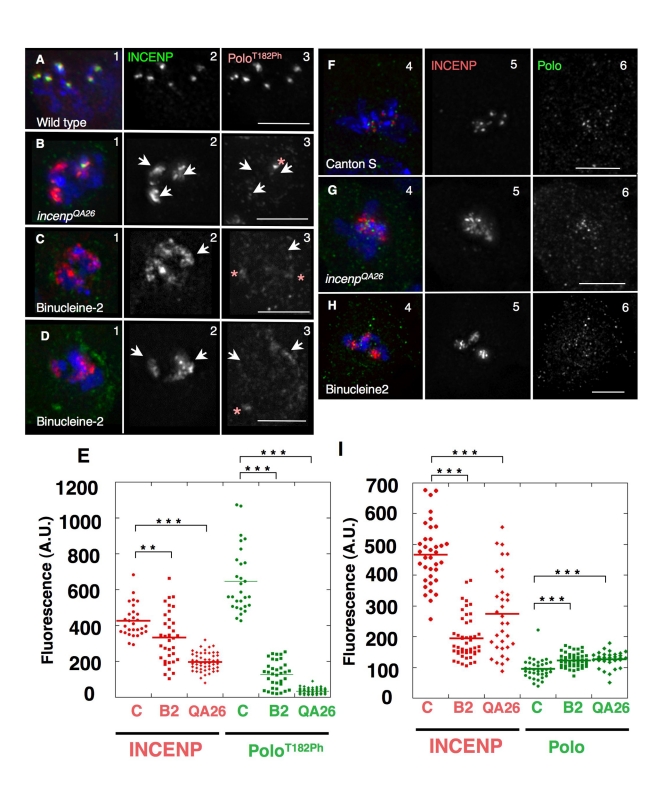
The centromeric activation of Polo in mitosis depends on INCENP and Aurora B in vivo. (A–E) Immunostaining analysis of the phosphorylation of Polo^T182^ in third instar neuroblasts of (A) wild type larvae (Canton-S), (B) *incenp ^QA26^* mutant larvae, and (C–D) wild type larvae treated with the Aurora B-specific inhibitor Binucleine 2. INCENP (2, green), Polo^T182Ph^ (3, red). Arrows point to INCENP blocks characteristic of the *incenp ^QA26^* and Binucleine-2 treatment phenotype. Asterisks indicate centrosomes. (E) Dot plot showing the quantification of INCENP/Polo^T182Ph^ signal intensity at the kinetochore (*t* test: *** *p*<0.0001; ** *p* = 0.003). Signal intensities for individual kinetochores were measured using the SoftWorx Data Inspector tool; average background was subtracted; data was plotted using KaleidaGraph software. (F–I) Levels of Polo kinase are not affected by defects in Incenp or Aurora B function (F) wild type larvae (Canton-S), (G) *incenp ^QA26^* mutant larvae, and (H) wild type larvae treated with the Aurora B-specific inhibitor Binucleine 2. INCENP (5, red), Polo (6, green). (I) Dot plot showing the quantification of INCENP/Polo signal intensity at the kinetochore (*t* test: *** *p*<0.0001). Signal intensities for individual kinetochores were measured using the SoftWorx Data Inspector tool; average background was subtracted; data were plotted using KaleidaGraph software.

We observed a strong signal of Polo^T182ph^ concentrated at kinetochores in wild-type mitotic neuroblasts (Figure 5A3). In third instar larval neuroblasts from the *incenp^QA26^* mutant, 27% of mitoses (*n* = 290) showed obvious defects in INCENP localization, with the protein spreading onto chromosome arms (Figures 5B2, S5E). This was never observed in wild-type neuroblasts (*n* = 303; Figures 5A, S5D). The *incenp^QA26^* mitotic phenotype (Figure S5E) resembles the Binucleine 2-induced phenotype, with INCENP dispersed in clumps on the chromosome arms in affected cells (Figure 4C,D; Figure S5B,C). Levels of Polo^T182ph^ at kinetochores were substantially reduced in *incenp^QA26^* mutant mitoses showing this characteristic *incenp* phenotype (Figure 5B3; Figure 5E). In contrast, overall levels of Polo at kinetochores remained similar to wild type (Figure 5G and I).

To test if Polo^T182^ phosphorylation requires Aurora B activity in vivo, we dissected whole brains, treated them with Binucleine 2, and processed them for immunostaining as above. After a 2-h incubation in 20 µM Binucleine 2, Histone H3^S10ph^ (a reporter for Aurora B activity) was undetectable in mitotic cells (unpublished data). Drug-treated neuroblasts also showed the characteristic dispersion of INCENP in clumps on chromosome arms (Figure 5C2,D2 arrowheads; Figure 5H).

As predicted, Polo^T182ph^ was virtually undetectable at kinetochores of Binucleine 2-treated neuroblasts, but remained readily observable at centrosomes (Figure 5C3,D3 asterisks). Total Polo levels at kinetochores remained similar to wild type in these cells. Thus, both Aurora B activity and INCENP are required for Polo T182 phosphorylation at kinetochores in mitotic Drosophila neuroblasts.

As independent confirmation that the CPC contributes to Polo regulation in vivo, we conducted a genetic experiment in a sensitized background. Tubulin-Gal4-driven overexpression of Polo mutated in a conserved destruction box (PoloΔdb—Figure S7) is semi-lethal at the pupal stage, suggesting that excessive Polo activity is detrimental to development. Interestingly, a decrease in the levels of functional INCENP rescued the semi-lethality of PoloΔdb-expressing flies. This was observed when one wild-type allele of *incenp* was replaced with either of the alleles *incenp ^EC3747^* or *incenp ^QA26^* (Figure S7). A heterozygous deletion removing Aurora B did not cause a significant rescue in the same assay, suggesting that INCENP may be the limiting CPC component for Polo regulation.

Together, these results confirm that the CPC contributes to the regulation of Polo function at kinetochores in vivo.

### The CPC Is Required for Plk1 Activation at Kinetochores in Human Cells

**Figure 6:**
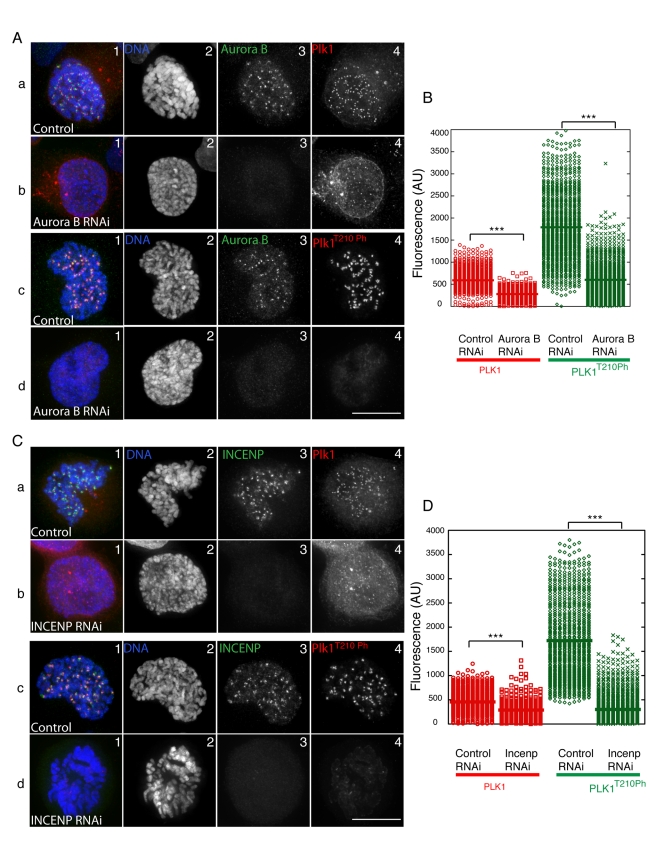
RNAi depletion of Aurora B or INCENP in human cells strongly reduces Plk1^T210Ph^ levels at kinetochores. (A) Control and Aurora B-depleted cells were fixed and immunostained with α-Aurora B (green), α-Plk1 (red), α-Plk1^T210Ph^ (red), and DNA (blue). (B) Quantification graph of Plk1 and Plk1^T210Ph^ levels at centromeres in Control and Aurora B-depleted cells. Fluorescence intensities are in Arbitrary Units (A.U.). (C) Control and INCENP-depleted cells were fixed and immunostained with α-INCENP (green), α-Plk1 (red), α-Plk1^T210Ph^ (red), and DNA (blue). Scale bar = 10 µm. (D) Quantification graph of Plk1 and Plk1^T210Ph^ levels at centromeres in Control and INCENP-depleted cells. Fluorescence intensities are in Arbitrary Units (A.U.) *t* test: *** *p*<0.0001.

Importantly, the regulation of Polo T-loop phosphorylation described above for Drosophila is conserved in human cells. We used siRNAs to deplete either Aurora B (Figure 6A) or INCENP (Figure 6C) in HeLa cells and subsequently measured the levels of total Plk1 and Plk1^T210Ph^ at kinetochores. Both INCENP and Aurora B depletion caused a dramatic reduction in Plk1^T210Ph^ levels in early mitosis (Figure 6Ad3,B,Cd3,D). Levels of Plk1 were also slightly reduced, confirming that the CPC is at least partly required for the stable localization of Plk1 to mammalian kinetochores [27]. Comparable results were obtained when we treated cells with the Aurora B kinase inhibitor ZM447439 (Figure S8), confirming that Aurora B activity is indeed required for the presence of Plk1^T210Ph^ at kinetochores in human cells.

Our results thus indicate that the CPC activates Polo kinase by T-loop phosphorylation at centromeres in both flies and humans.

## Discussion

Coordination of Polo and Aurora B activity at kinetochores is critical in early mitosis, as the two kinases play potentially antagonistic but complementary roles in regulating kinetochore-microtubule interactions. Aurora B is essential for the correction of aberrant attachments [13]–[15], and indeed, tethering Aurora B too close to kinetochores interferes with the formation of stable attachments [16]. In contrast, Plk1 activity is required for initial stabilisation of microtubule attachments to kinetochores [7]–[9]. We suggest that interactions with INCENP may provide a mechanism to coordinate the activities of these two essential kinases during early mitosis.

Recent studies suggest that Plk1 is activated at centrosomes when its T-loop (T210) is phosphorylated by Aurora A kinase–Bora, and that this promotes the G2/M transition upstream of Cdk1 [21],[22], although Polo activity is not required for mitotic entry ([3]; this paper–Figure 3). How Plk1 is activated at kinetochores remained an important unsolved question. Our present results show that Aurora B and INCENP, which are concentrated at inner centromeres [43],[44], function there to activate Polo by phosphorylating its T-loop.

Plk1 recruitment to centromeres in late G2 has been variously proposed to be mediated by Bub1 [45], INCENP [27], and BubR1 [7]. Another report implicated the self-primed interaction of Plk1 with PBIP1/CENP-U [46]. This could potentially explain why Plk1 activity is reportedly required for its localisation to kinetochores in human cells [47].

Our RNAi studies confirmed that Plk1 is partially dependent on the CPC for its centromeric localization in human cells. However, this appears not to be the case in Drosophila, where Polo is present at centromeres before NEB, at a time when INCENP is not yet concentrated at inner centromeres and before Polo^T182ph^, the active form of the kinase, is detected there. Indeed, we observed no significant decrease in kinetochore-associated Polo levels after INCENP RNAi in Drosophila cells.

**Figure 7:**
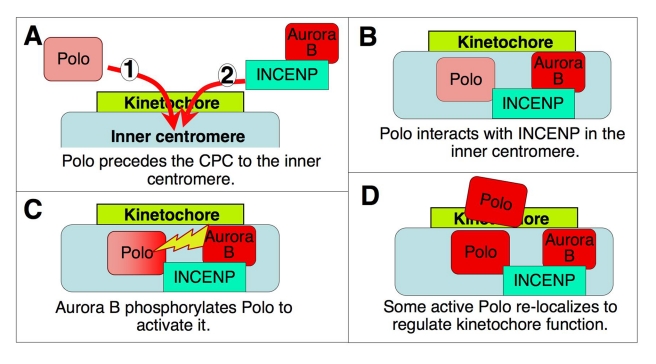
Model for the interactions between the CPC and Polo kinase at the centromere/kinetochore. See text for details.

Although Polo targeting to kinetochores is independent of the CPC in Drosophila, its activation there does require the CPC with active Aurora B. Our data suggest that INCENP binding to Polo facilitates its subsequent activation by Aurora B kinase (Figure 7B,C). Indeed, INCENP and Polo interact physically in vitro and co-immunoprecipitate in mitotic cell extracts. Although most centromeric Polo kinase is concentrated in the outer kinetochore in prophase and prometaphase, active Polo (Polo^T182ph^) is also found in inner centromeres, where it overlaps with INCENP as confirmed by a proximity ligation assay (PLA).

A range of evidence presented here suggests that Aurora B is the upstream kinase responsible for Polo kinase activation at centromeres. Firstly, Aurora B phosphorylates Polo at Thr182 in vitro. Secondly, RNAi depletion of INCENP or Aurora B, but not Aurora A, reduces levels of active Polo^T182ph^ at kinetochores. Thirdly, tissue culture cells and third larval instar neuroblasts treated with a specific inhibitor of Drosophila Aurora B kinase show decreased levels of Polo^T182ph^ at kinetochores. In all of the preceding experiments, Polo^T182ph^ levels are affected at kinetochores but not at centrosomes, where Polo is presumably activated by Aurora A [21],[22]. Importantly, this involvement of Aurora B in Polo activation at centromeres discovered in Drosophila is conserved for Plk1 in human cells.

Our results suggest a model for interactions between Polo kinase and the CPC at centromeres (Figure 7). In Drosophila cells, Polo targets to centromeres before the CPC is recruited by Survivin binding to histone H3T3ph [17]–[19]. At inner centromeres of chromosomes whose kinetochores are not under tension, Polo now binds to INCENP. This promotes Polo kinase activation, as Aurora B phosphorylates Polo^T182^. We suggest that interactions with INCENP link the two complementary kinase activities, thereby potentially creating a microtubule attachment/detachment cycle at kinetochores. Such a cycle would not be possible without a balancing phosphatase activity, and PP2A-B56 has recently been shown to oppose both Aurora B and Plk1 activities at kinetochores to promote stable attachments [48].

At metaphase, when chromosomes are bioriented and under tension, the CPC and Polo kinase exhibit only a partial overlap. A weakening of the INCENP/Polo PLA signals in metaphase suggests that Polo may be released from INCENP after its activation—possibly moving to the outer kinetochore (Figure 7D). During metaphase, the CPC localizes in the inner centromere, stretching between sister kinetochores, whereas Polo and Polo^T182ph^ concentrate mainly at the kinetochores. This separation may be necessary to allow Polo-mediated stabilisation of kinetochore-microtubule attachments. The coordinated activities of both kinases at kinetochores and their tension-mediated separation might facilitate a dynamic equilibrium between attached and unattached kinetochores, selectively stabilizing proper chromosome attachments.

In summary, our results reveal that INCENP and Aurora B are responsible for Polo kinase activation at centromeres but not at centrosomes during mitosis. These experiments support the hypothesis that INCENP acts as a scaffold integrating the cross-talk between these two important mitotic kinases [26].

## Materials and Methods

### Drosophila Strains

Fly strains were grown at 25°C in standard Drosophila medium. The following stocks were used: Canton-S; *incenp^QA26^/SM6a, incenp^EC3747^/SM6a, Tubulin Gal-4/TM3*. *UASp-POLO*Δ*DB-MYC* transgenic flies were generated by BestGene Inc. Immunostaining of testes and larval neuroblasts was performed as described previously [49].

### Antibodies

Primary antibodies and dilutions for immunofluorescence analysis were as follows: mouse monoclonal B512 anti-αTubulin (SIGMA, 1∶2,000); Rabbit Polyclonals Rb-801, Rb-803 [41], 1∶500); mouse monoclonal anti-PhosphoT210 Plk1 (Abcam ab39068, 1∶100); mouse monoclonal anti-Polo Mab294 (kindly provided by A. Tavares and David Glover,1∶100); rabbit polyclonal anti-Aurora A (1∶100) and anti-Aurora B (1∶500) [50],[51]; and monoclonal anti-Myc 9E10 (Santa Cruz). Secondary antibodies were obtained from Jackson Immunoresearch.

### Drosophila Cell Culture, dsRNAi, Drug Treatment, and Immunofluorescence

The AC5-Polo-GFP cell line was described previously [52], and the AC5-Aurora B-GFP, AC5-Polo-Myc, AC5-Polo-T182A-Myc, and AC5-Polo-T182D-Myc stable cell lines were generated following the same protocol. Cell lines were grown in Express-Five medium (GIBCO) containing 20 µg/ml blasticidin.

Cells were treated with either DMSO or 20 µM Binucleine-2 for 2 to 4 h before being processed for immunostaining as described previously [41]. For experiments shown in Figures 3, 4F,G, and S6, 1.2×10^6^ D-Mel2 cells were transfected in 6-well plates with 20 µg of dsRNA using Transfast reagent (Promega). Cells were analysed 3 or 4 d later by immunofluorescence and immunoblotting. The control dsRNA was generated against the sequence of the bacterial Kanamycin resistance gene. For experiments shown in Figures 3, 4G, and S6A, cells were seeded on coverslips and treated for 10 s in BRB-80+0.1% NP-40 before a 20 min fixation in BRB-80+4% formaldehyde. Cells were then permeabilized for 10 min in BRB-80+0.1% Triton X-100 and blocked for 1 h in PBS+0.1% Tween20+1% BSA. Primary antibodies were diluted in PBS+0.1% Tween20+1% BSA and incubated overnight at 4°C. Secondary antibodies were incubated 2 h at room temperature. Coverslips were mounted with Vectashield+DAPI. Images were taken using an AxioImager epifluorescence microscope.

### Proximity Ligation Assay (PLA)

Proximity Ligation Assay was performed using Duolink QL (Olink, Uppsala, Sweden) following the manufacturer's protocol. Duolink anti-rabbit plus probe, anti-mouse minus probe, and anti-rat minus probe were used. The following antibody pairs were used for the assay: Rabbit polyclonal anti-Incenp Rb801 [41], 1∶500/mouse monoclonal anti-Polo Mab294 (kind gift of A. Tavares,1∶100); Rabbit polyclonal anti-Incenp Rb801 ([41], 1∶500)/mouse monoclonal anti-PhosphoT210Plk1 (Abcam,1∶100); Rat monoclonal anti-Incenp (Kind gift of Kim McKim, 1∶300)/Rabbit polyclonal anti-Aurora B 963 ([41],1∶500); Rabbit polyclonal anti-Incenp Rb801 ([41], 1∶500)/mouse monoclonal anti-GFP (Roche, 1∶500); Rabbit polyclonal anti-Incenp Rb801 ([41], 1∶500)/mouse monoclonal anti-γTubulin (Sigma 1∶50); and Rabbit polyclonal anti-CID (a gift from S. Henikoff,1∶500)/mouse monoclonal anti-Polo Mab294 (1∶100). In each experiment a negative control using only one antibody of each pair was included.

For each antibody pair, exponentially growing DMel-2 cells were seeded on Con-A treated coverslips and fixed for immunostaining as described previously [41]. After overnight incubation with primary antibody at 4°C, half of the samples were processed following the normal immunostaining protocol [41] and the other half was used for the PLA assay.

Imaging was performed using Olympus IX-71 microscope controlled by Delta Vision SoftWorx (Applied Precision, Issequa, WA, USA). Image stacks were deconvolved, quick-projected, and saved as tiff images to be processed using Adobe Photoshop. Linescans were generated using Image-Pro software.

### Plasmids and Mutagenesis

*POLO-T182A*, *T182D*, and *POLOΔDB* (*R309A*, *L312A*) were generated in the *pDONR221* (Invitrogen) using QuickChange (Stratagene). The expression vectors *pAC5-POLO-MYC*, *pAC5-POLO-T182A-MYC*, *pAC5-POLO-T182D-MYC*, and *pUASp-POLOΔDB-MYC* were generated by Gateway recombination (Invitrogen) of *pDONR-*based entry clones into *pDEST-AC5-Cterm-MYC* and *pDEST-UASp-Cterm-MYC*, respectively. *POLO-WT* and *POLO-T182A* were cloned into *pETDuet* for expression as N-terminal fusions with a *HIS* tag at the MCS1 position. *Aurora B* was cloned into *pDONR221*, which was then recombined into *pDEST-AC5-Cterm-GFP* to generate *pAC5-AURORA B-GFP*.

### In Vitro Binding Assays

GST tagged full-length Drosophila Incenp was expressed in bacteria (BL21) and purified on Glutathione sepharose beads as described previously [42]. Polo, Aurora B, and Luciferase were in vitro translated using a coupled transcription/translation reticulocyte lysate system (Promega's TNT system). Binding buffer—50 mM Tris pH 7.5, 10 mM MgCl_2_, 1 mM EGTA, 1 mM DTT, 0.1% Triton X-100, 0.5 mM PMSF, and 1 mg/ml CLAP.

### Immunoprecipitation

Mouse anti-GFP (Roche) and mouse IgG (Abcam)—negative control—antibodies were crosslinked to protein G Dynabeads (Invitrogen) at 0.5 µg of antibody/1 µl of beads. Exponentially growing D-Mel2 cells were lysed on ice in lysis buffer (for Polo-GFP cell line: 40 mM Tris-Cl [pH 7.5], 100 mM NaCl, 1 mM PMSF, 1 mM DTT, 10 mM EGTA, 1% Triton-X-100, and protease inhibitor cocktail (Roche, UK); for GFP-Aurora B cell line: 50 mM Tris-Cl (pH 8.0), 150 mM NaCl, 1 mM EDTA, 1% NP40, 0.5% deoxycholate, and protease inhibitor cocktail -Roche). Cell lysates were separately incubated with either mAb anti-GFP or mouse IgG bound to Dynabeads protein G for 1 h at 4°C. Samples were spun down, then washed first with lysis buffer, and then twice with wash buffer (40 mM Tris-Cl [pH 7.5], 100 mM NaCl, 1 mM PMSF, 10 mM EGTA, 0.1% Triton-X-100, protease inhibitor cocktail, Roche, UK). Finally the beads were boiled in Laemmli sample buffer. All samples were subjected to SDS-PAGE and analyzed by immunoblotting as described before.

### Kinase Assay

HIS-Polo and HIS-Polo-T182A were expressed in BL21 bacteria from *pETDuet*-based constructions (see above). Protein purification was done using Talon resin (Clontech) and purified proteins were stored on the resin at −80°C.

For the kinase assay, HIS-Polo and HIS-Polo-T182A on the resin were incubated with Drosophila Aurora B in complex with INCENP_645–755_ [39] for 15 min at 30°C in 20 mM K-HEPES pH 7.5, 2 mM MgCl_2_, 1 mM DTT, 500 mM ATP, 5 mCi ^32^P-g-ATP. Reactions were initiated by the combination of the substrate-bound resins to a fixed volume of a master mix containing all other reagents. Reactions were stopped by the addition of Laemmli sample buffer. Samples were separated by SDS-PAGE and transferred to nitrocellulose. Quantitative, sub-saturation measurements of radioactivity and Polo Western blot signals were obtained using a PhosphorImager and a Typhoon luminescence reader, respectively.

### siRNAi, Drug Treatments, Immunofluorescence, and Imaging in Human Cells

HeLa Kyoto were grown in Dulbecco's modified Eagle's medium, supplemented with 10% foetal calf serum, 0.2 mM l-Glutamine, 100 U/ml penicillin, and 100 µg/ml streptomycin.

RNAi experiments were performed using annealed siRNA oligos (Qiagen) diluted in serum free OptiMem and transfected using HiPerFect reagent (Qiagen) according to the manufacturer's protocol. HeLa cells were seeded on coverslips at a density of 1×10^5^ cells/ml and diluted siRNA was added to cells so that the final concentration of siRNA was 40 nM. Coverslips were fixed at 48 h. For control transfections non-silencing random scramble siRNA oligos were used at the same concentration. The full sequences of siRNA oligos used are as follows: for Aurora B siRNA, 5′-AACGCGGCACTTCACAATTGA-3′; for INCENP siRNA 5′-AGATCAACCCAGATAACTA-3′. For drug treatments, ZM447439 (Tocris Bioscience) or DMSO as control were added to the cells at the concentration of 3 µM for 1 h.

All fixation, permeabilisation and immunostaining were performed at room temperature, as previously described [53]. Anti-Aurora B rabbit polyclonal at 1∶100 (Abcam, ab2245), anti-INCENP rabbit polyclonal at 1∶100 (Upstate), anti-Plk1 1∶100 mouse monoclonal (Abcam), anti-P-Plk1 (T210) 1∶100 mouse monoclonal (Abcam), and anti-phospho-Histone H3 (Ser10) rabbit polyclonal (Upstate). All affinity purified donkey secondary antibodies (labelled either with FITC, TRITC, or CY5) were purchased from Jackson Immunoresearch.

Quantification of Plk1 and Plk1^T210Ph^ on approximately 1,000 centromeres per condition was carried out as follows: Deconvolved images were imported into OMERO [54] and segmentation of centromere foci (ACA, Cy5, reference channel) performed using Otsu segmentation implemented in Matlab. Masks stored in OMERO were then used to calculate intensities, and output into comma-separated value file for plotting in Excel.

## Supporting Information

Figure S1 Localization of Polo, INCENP, and tubulin during mitosis in Drosophila cultured cells. (A) Prophase, Polo on centrosomes and kinetochores. No Incenp at centromeres. (B) Early prometaphase. (C) Late prometaphase. (D) Metaphase. Merged panel shows DNA (blue), Polo (green), and Tubulin (red). High magnification insets show INCENP in blue. Scale bar = 5 µm.
(TIF)

Figure S2 Relative localizations of Polo and Aurora kinases during mitosis in Drosophila cultured cells. (A–B) Aurora A (red) and Polo (green) colocalize at centrosomes throughout mitosis (arrowheads). (C–D) Aurora B (red) and Polo (green) both localize at the centromere/kinetochores region in early mitosis. Scale bar = 5 µm.
(TIF)

Figure S3 Characterization of the anti-Polo^T182Ph^ antibody by immunofluorescence. (A–D) Distribution of Polo kinase (green) and the active form of the kinase (Polo^T182Ph^, red) during mitosis. (A) Polo/Polo^T182Ph^ are present at centrosomes at a time in which Polo—but not Polo^T182Ph^—accumulates at kinetochores. (B) Polo/Polo^T182Ph^ colocalize at kinetochores and centrosomes in metaphase and (C–D) also at the central spindle at anaphase and telophase. (E) Specificity of the antibody against Polo^T182Ph^ in immunofluorescence. D-Mel cells were treated with Polo dsRNA or not for 60 h, fixed, and stained for pT182-Polo and alpha-tubulin or CENP-A (centromere). The pT182-Polo stainings at centromeres/KTs and centrosomes are largely abolished. pT182-Polo stainings of the centrosomes and the midbody in cytokinesis were strongly diminished, but never completely abolished, probably because cells that could complete mitosis were those for which Polo depletion was only partial. In addition, we always observed a non-specific staining of unknown nature at or near the DNA, which remained visible during mitosis in Polo-depleted cells more than in control cells.
(TIF)

Figure S4 Colocalization of INCENP/Polo/Polo^T182Ph^ changes through mitosis. High magnification images of kinetochores in (A–C) late prophase/early prometaphase and (D–F) metaphase in cultured cells. INCENP, red; Polo, green; and Polo^T182Ph^, blue). Linescans show signal intensity across a kinetochore/inner centromere/kinetochore line. The graph profile shows specific accumulation of Polo^T182Ph^ at the inner centromere at the earlier stages of mitosis; at later stages the Polo^T182Ph^ graph resolves in two clear peaks closer to the kinetochore.
(TIF)

Figure S5 CPC localization is similar in Binucleine-2 treated cells and *incenp* mutants in prometaphase. DMel-2 cells treated with (A) DMSO or (B–C) Binucleine-2 and stained for INCENP (green) and Aurora B (red). (B) Prometaphase. (C) Binucleate cell. (D) Wild type and (E) *incenp^QA26^* mutant neuroblasts stained for INCENP (red) and Histone3^Ser10Ph^.
(TIF)

Figure S6 RNAi depletion of Aurora B or INCENP does not reduce Polo^T182Ph^ levels at centrosomes. (A) Cells were treated with the indicated dsRNAs for 3 d and Polo^T182Ph^ was detected by immunofluorescence. Levels of Polo^T182Ph^ at individual centrosomes in prometaphase and metaphase cells were measured using Image J as in Figure 4G. (B) Immunoblots showing levels of protein depletion after dsRNA treatments.
(TIF)

Figure S7 A decrease in INCENP activity partially rescues the lethality induced by a gain of Polo function. (A) A conserved destruction box in Polo was mutated in PoloΔdb. (B) Female flies heterozygous for a *UASp-PoloΔdb-Myc* transgene and strongly hypomorphic *incenp* alleles were crossed to males heterozygous for the *Tubulin-Gal4* driver. (C) Expression of this *UASp-PoloΔdb-Myc* transgene driven by *Tubulin-Gal4*, is semi-lethal. Progeny flies combining the *incenp* allele, *UASp-PoloΔdb-Myc* transgene, and the *Tubulin-Gal4* driver were identified by the absence of phenotypic markers from balancer chromosomes. The number of flies obtained relative to the expected numbers (one-fourth of the total progeny) is shown for each *incenp* genotype. *N*, total numbers of progeny obtained and scored from three vials for each cross. The only definitely null aberrations that we could obtain were large deficiencies that could not be tested because they interacted with balancer chromosomes in our crosses; therefore, it remains formally possible, although unlikely, that both *incenp* alleles tested here have antimorphic effects.
(TIF)

Figure S8 Aurora B activity is required for activation of Plk1 at centromere/kinetochores in human cells. (A) HeLa cells treated with DMSO or ZM447439 immunostained for Histone H3 P-Ser10 (green), Plk1 (red), or Plk1^T210Ph^ (red) and DNA (blue). Scale bar = 10 µm. (B) Quantification graph of Plk1 and Plk1^T210Ph^ levels at centromeres in Control and ZM447439 treated cells. Fluorescent intensities are in Arbitrary Units (A.U.). *t* test: *** *p*<0.0001.
(TIF)

## Abbreviations

CPC: chromosomal passenger complex
KMN: KNL-1/Mis12/Ndc80
KT-MT: kinetochore-microtubule
NEB: nuclear envelope breakdown
OA: okadaic acid
PLA: proximity ligation assay
