# Correction: The Chromosomal Passenger Complex Activates Polo Kinase at Centromeres

**DOI:** 10.1371/annotation/7832f573-e0d9-465f-b5d4-0ac1014b6112

**Published:** 2012-02-14

**Authors:** Mar Carmena, Xavier Pinson, Melpi Platani, Zeina Salloum, Zhenjie Xu, Anthony Clark, Fiona MacIsaac, Hiromi Ogawa, Ulrike Eggert, David M. Glover, Vincent Archambault, William C. Earnshaw

Figure S5 is a duplicate of Figure S7. Please view the correct Figure S5 here: 
