# Supplementary figures and images for: The Chromosomal Passenger Complex Activates Polo Kinase at Centromeres
Source: PLoS Biol. 2012 Jan 24;10(1):e1001250. doi: 10.1371/journal.pbio.1001250 (PMC3265468; doi:10.1371/journal.pbio.1001250)

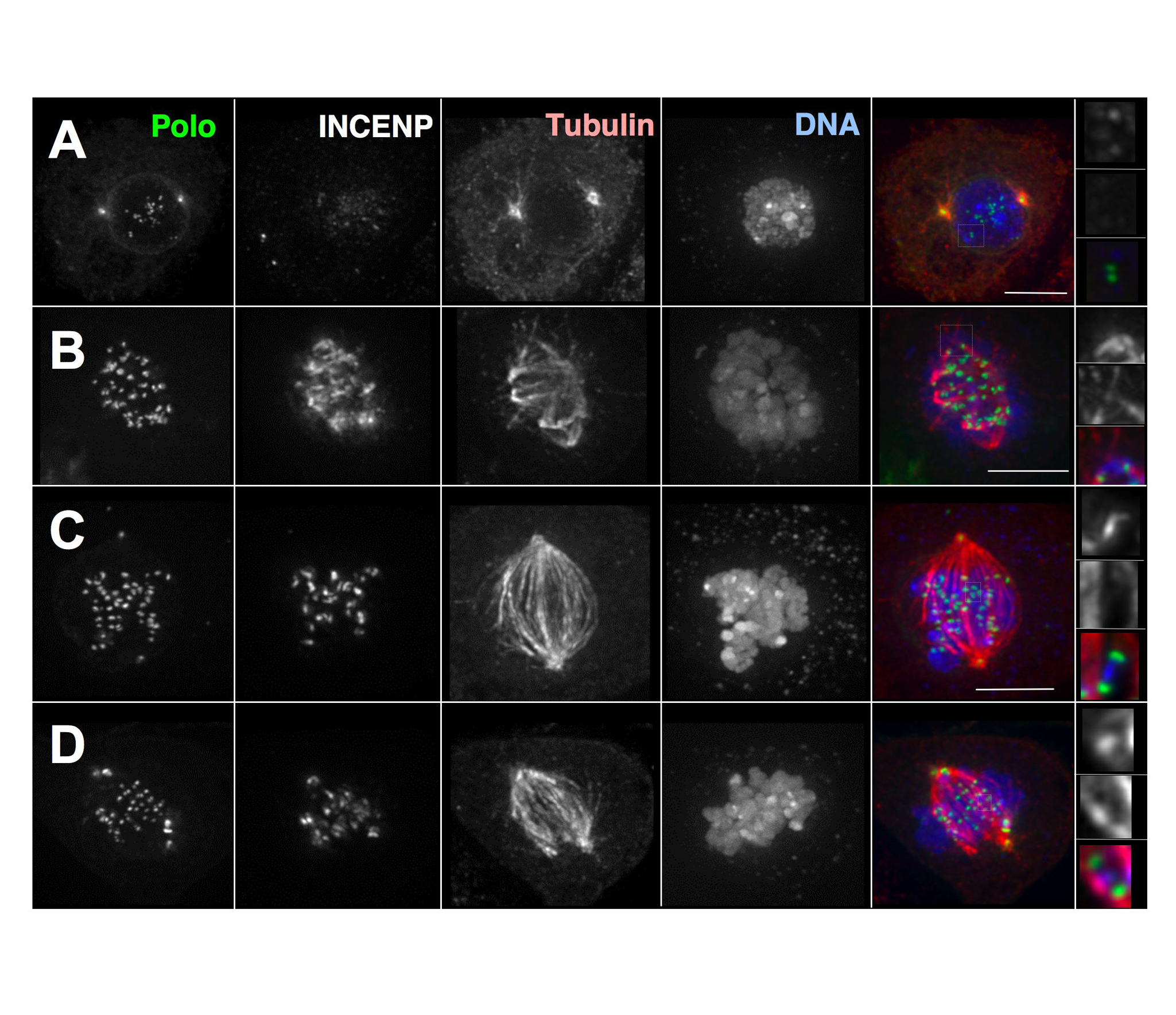

Supplement: Figure S1 — Localization of Polo, INCENP, and tubulin during mitosis in Drosophila cultured cells. (A) Prophase, Polo on centrosomes and kinetochores. No Incenp at centromeres. (B) Early prometaphase. (C) Late prometaphase. (D) Metaphase. Merged panel shows DNA (blue), Polo (green), and Tubulin (red). High magnification insets show INCENP in blue. Scale bar = 5 µm. (TIF) [file pbio.1001250.s001.tif]

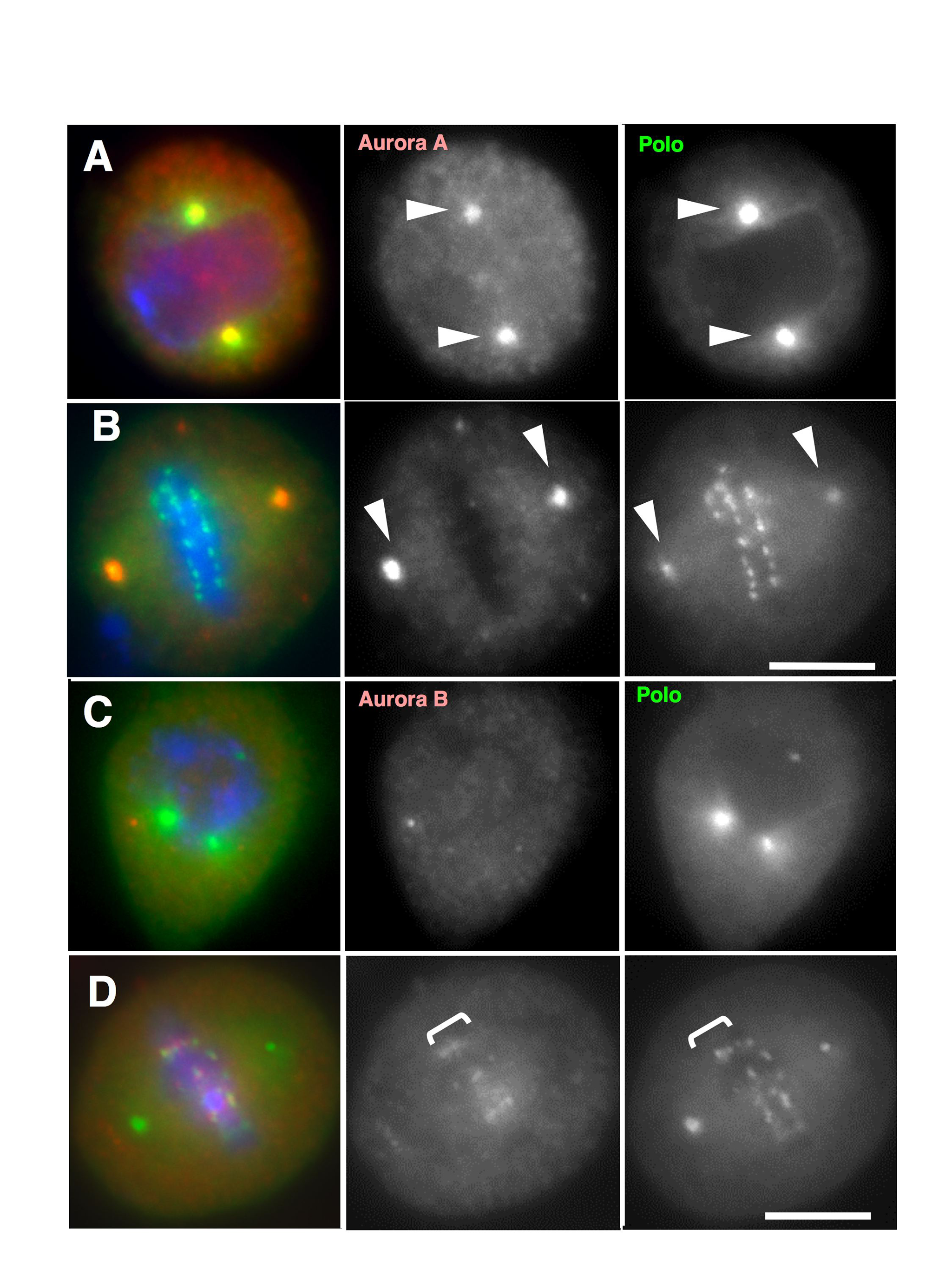

Supplement: Figure S2 — Relative localizations of Polo and Aurora kinases during mitosis in Drosophila cultured cells. (A–B) Aurora A (red) and Polo (green) colocalize at centrosomes throughout mitosis (arrowheads). (C–D) Aurora B (red) and Polo (green) both localize at the centromere/kinetochores region in early mitosis. Scale bar = 5 µm. (TIF) [file pbio.1001250.s002.tif]

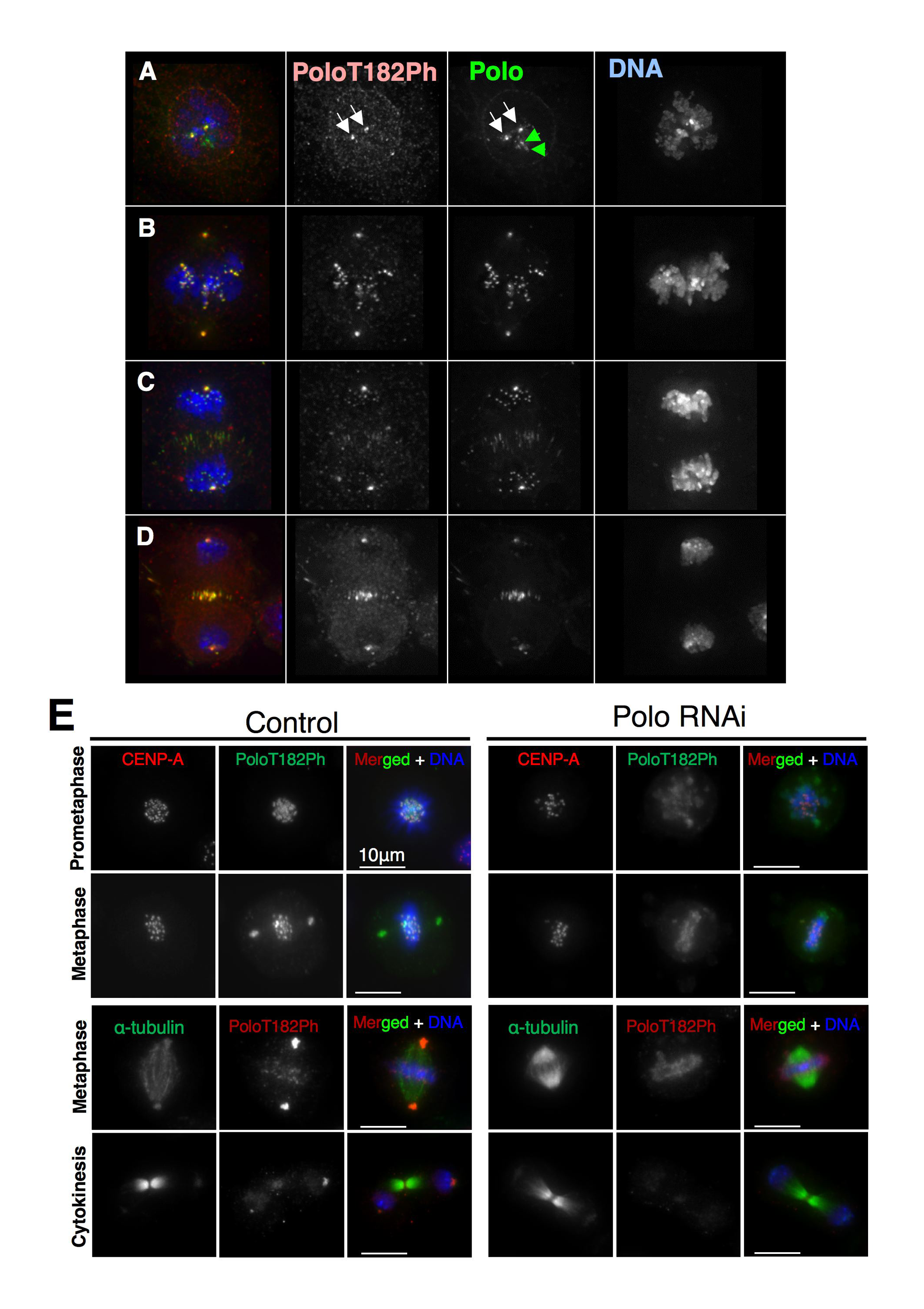

Supplement: Figure S3 — Characterization of the anti-PoloT182Ph antibody by immunofluorescence. (A–D) Distribution of Polo kinase (green) and the active form of the kinase (PoloT182Ph, red) during mitosis. (A) Polo/PoloT182Ph are present at centrosomes at a time in which Polo—but not PoloT182Ph—accumulates at kinetochores. (B) Polo/PoloT182Ph colocalize at kinetochores and centrosomes in metaphase and (C–D) also at the central spindle at anaphase and telophase. (E) Specificity of the antibody against PoloT182Ph in immunofluorescence. D-Mel cells were treated with Polo dsRNA or not for 60 h, fixed, and stained for pT182-Polo and alpha-tubulin or CENP-A (centromere). The pT182-Polo stainings at centromeres/KTs and centrosomes are largely abolished. pT182-Polo stainings of the centrosomes and the midbody in cytokinesis were strongly diminished, but never completely abolished, probably because cells that could complete mitosis were those for which Polo depletion was only partial. In addition, we always observed a non-specific staining of unknown nature at or near the DNA, which remained visible during mitosis in Polo-depleted cells more than in control cells. (TIF) [file pbio.1001250.s003.tif]

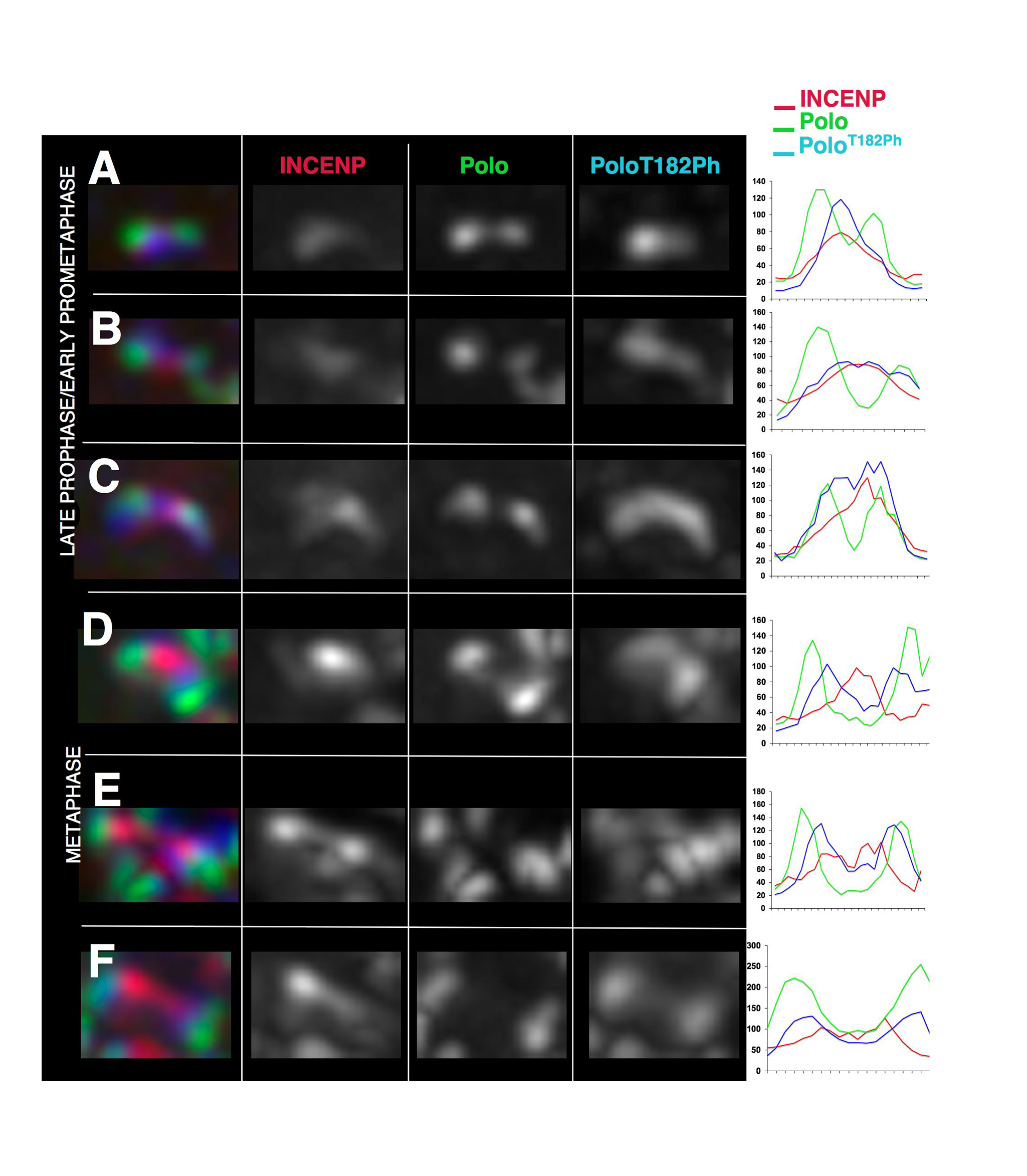

Supplement: Figure S4 — Colocalization of INCENP/Polo/PoloT182Ph changes through mitosis. High magnification images of kinetochores in (A–C) late prophase/early prometaphase and (D–F) metaphase in cultured cells. INCENP, red; Polo, green; and PoloT182Ph, blue). Linescans show signal intensity across a kinetochore/inner centromere/kinetochore line. The graph profile shows specific accumulation of PoloT182Ph at the inner centromere at the earlier stages of mitosis; at later stages the PoloT182Ph graph resolves in two clear peaks closer to the kinetochore. (TIF) [file pbio.1001250.s004.tif]

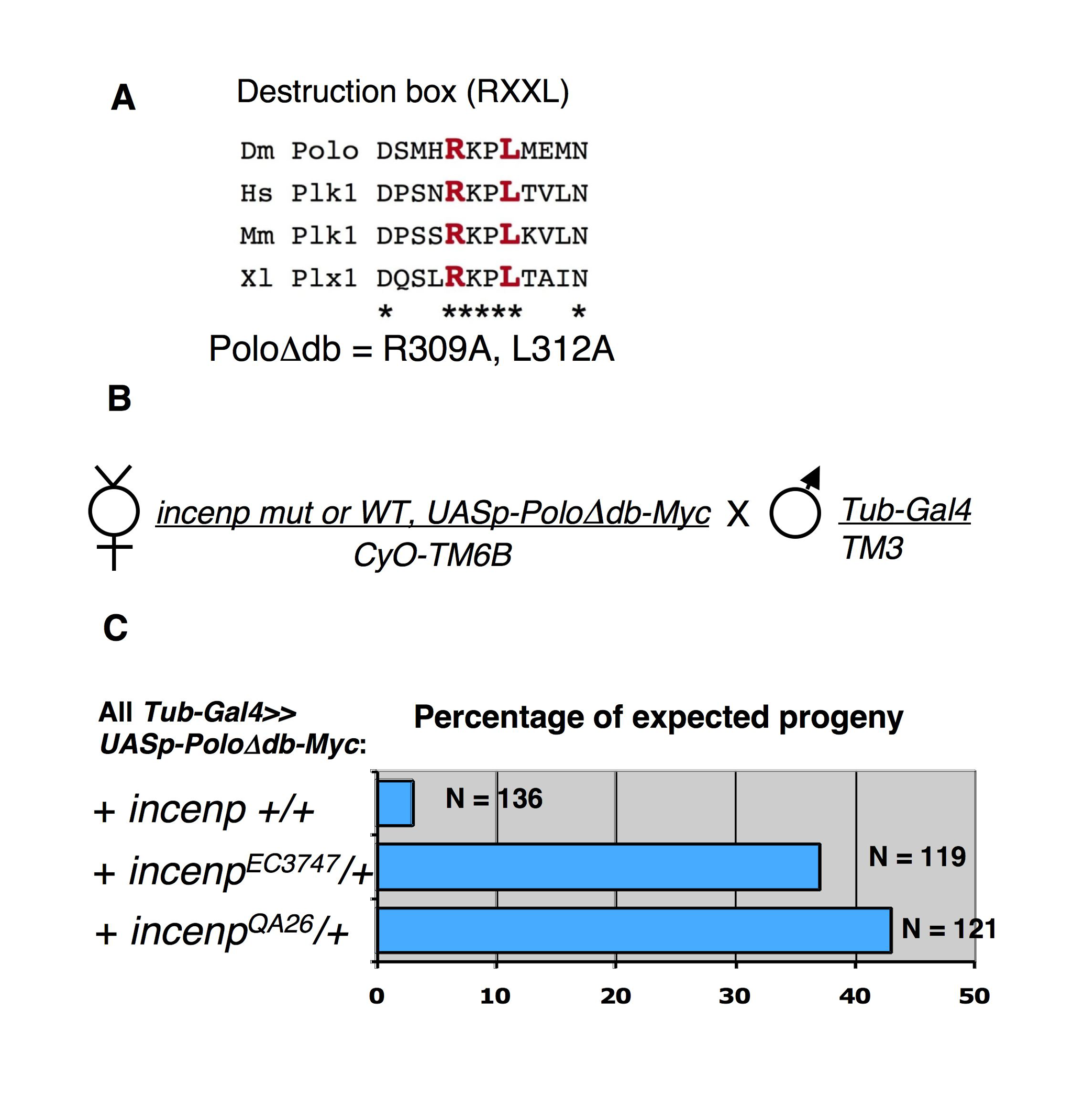

Supplement: Figure S5 — CPC localization is similar in Binucleine-2 treated cells and incenp mutants in prometaphase. DMel-2 cells treated with (A) DMSO or (B–C) Binucleine-2 and stained for INCENP (green) and Aurora B (red). (B) Prometaphase. (C) Binucleate cell. (D) Wild type and (E) incenpQA26 mutant neuroblasts stained for INCENP (red) and Histone3Ser10Ph. (TIF) [file pbio.1001250.s005.tif]

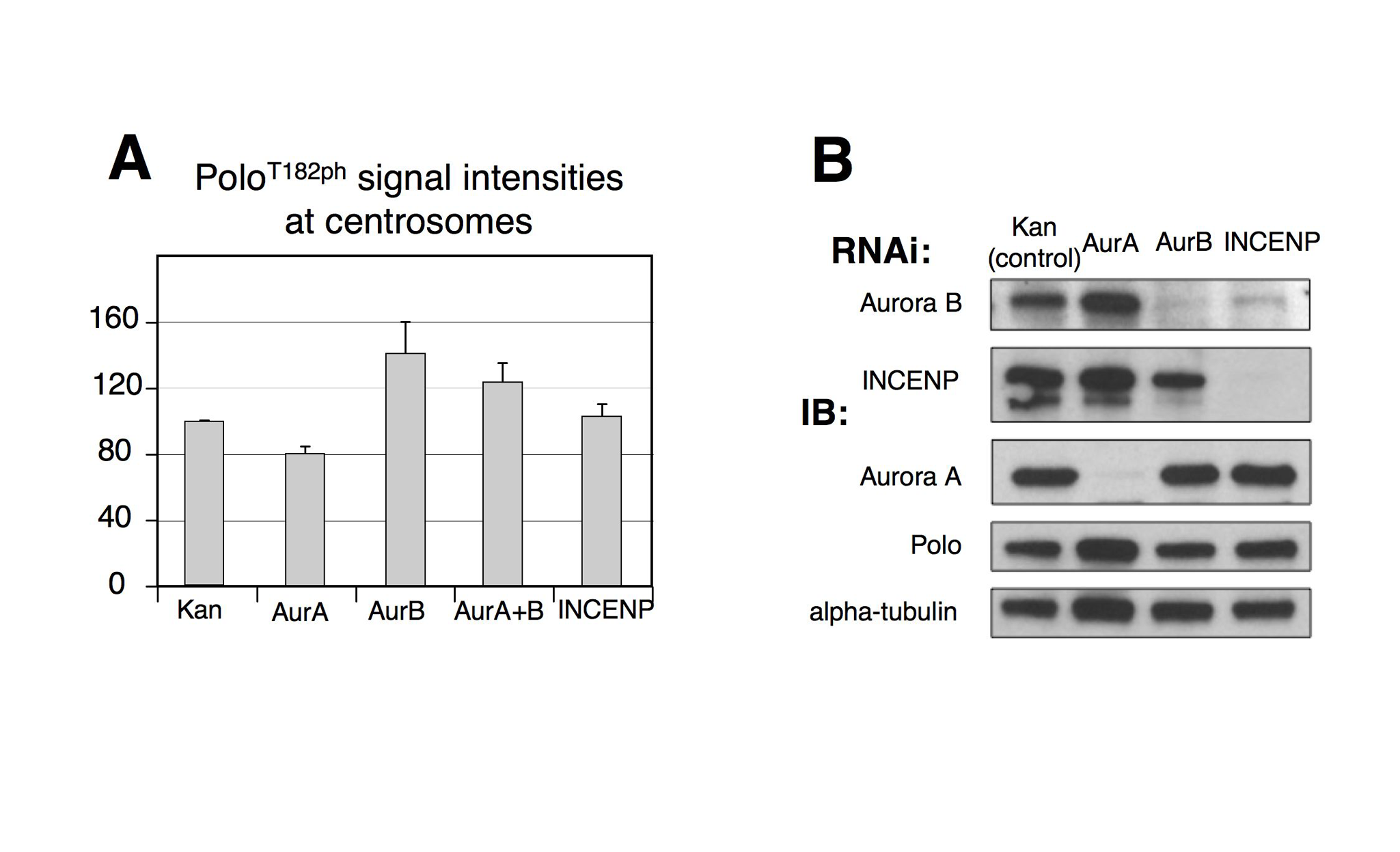

Supplement: Figure S6 — RNAi depletion of Aurora B or INCENP does not reduce PoloT182Ph levels at centrosomes. (A) Cells were treated with the indicated dsRNAs for 3 d and PoloT182Ph was detected by immunofluorescence. Levels of PoloT182Ph at individual centrosomes in prometaphase and metaphase cells were measured using Image J as in Figure 4G. (B) Immunoblots showing levels of protein depletion after dsRNA treatments. (TIF) [file pbio.1001250.s006.tif]

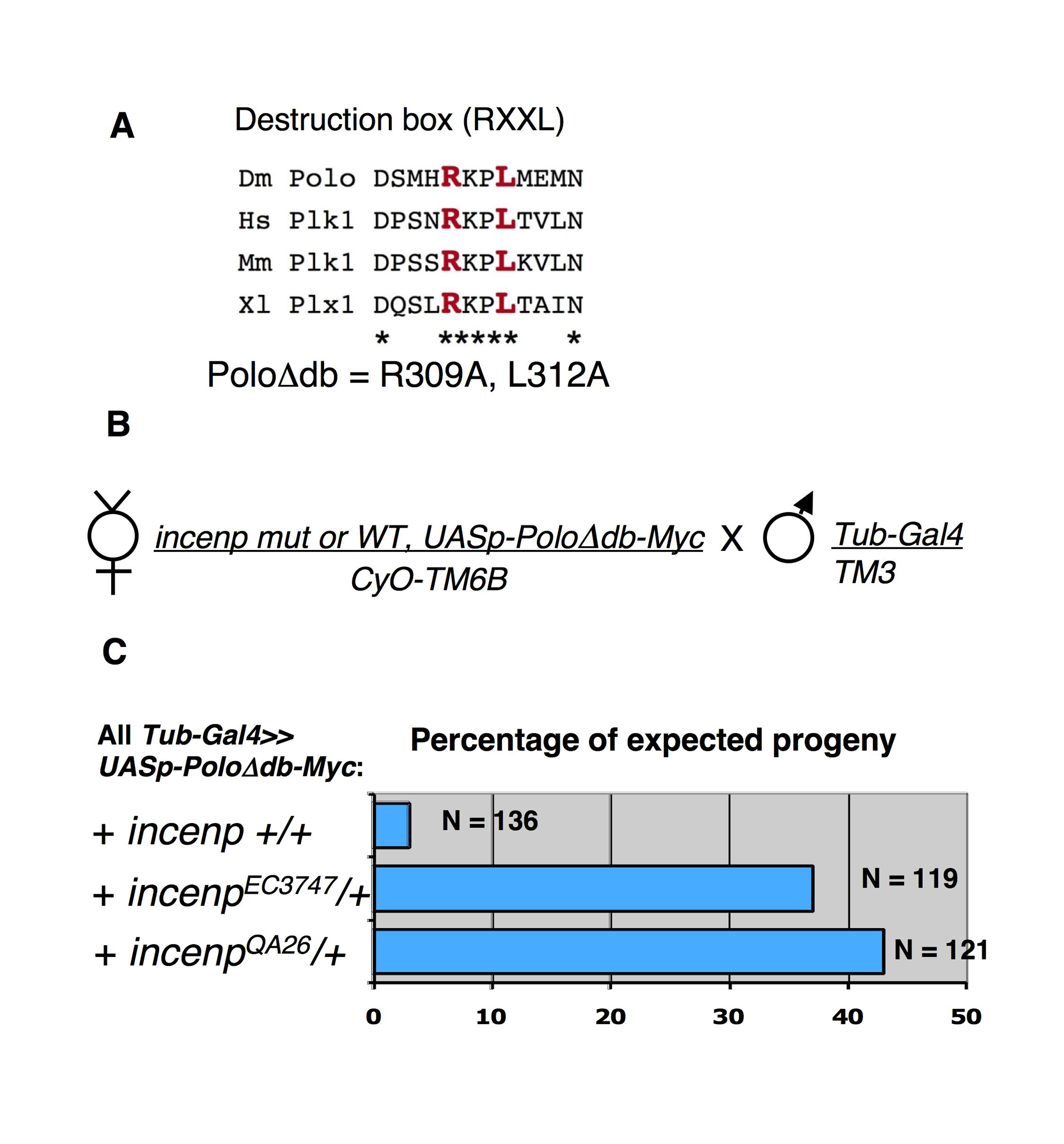

Supplement: Figure S7 — A decrease in INCENP activity partially rescues the lethality induced by a gain of Polo function. (A) A conserved destruction box in Polo was mutated in PoloΔdb. (B) Female flies heterozygous for a UASp-PoloΔdb-Myc transgene and strongly hypomorphic incenp alleles were crossed to males heterozygous for the Tubulin-Gal4 driver. (C) Expression of this UASp-PoloΔdb-Myc transgene driven by Tubulin-Gal4, is semi-lethal. Progeny flies combining the incenp allele, UASp-PoloΔdb-Myc transgene, and the Tubulin-Gal4 driver were identified by the absence of phenotypic markers from balancer chromosomes. The number of flies obtained relative to the expected numbers (one-fourth of the total progeny) is shown for each incenp genotype. N, total numbers of progeny obtained and scored from three vials for each cross. The only definitely null aberrations that we could obtain were large deficiencies that could not be tested because they interacted with balancer chromosomes in our crosses; therefore, it remains formally possible, although unlikely, that both incenp alleles tested here have antimorphic effects. (TIF) [file pbio.1001250.s007.tif]

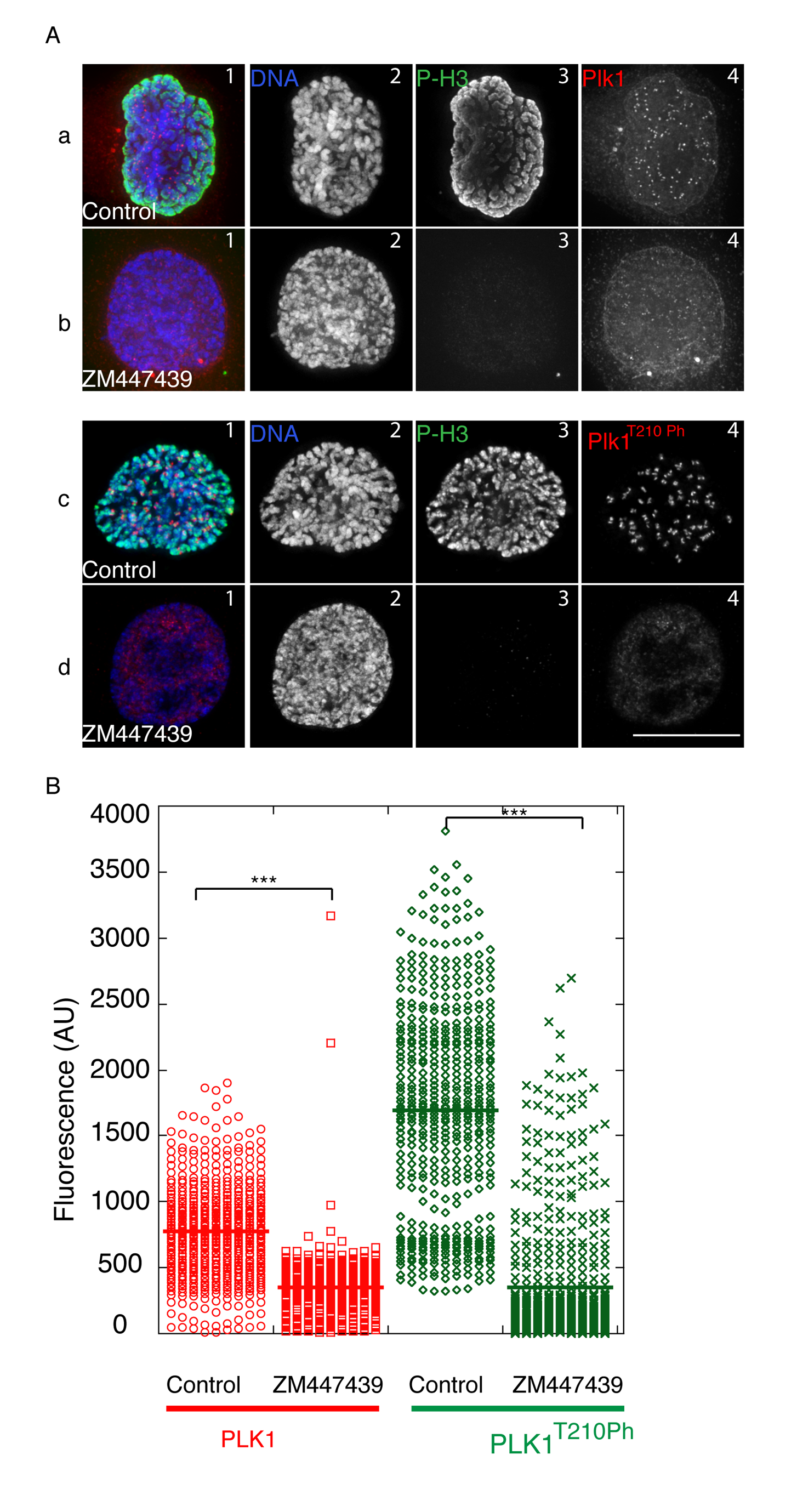

Supplement: Figure S8 — Aurora B activity is required for activation of Plk1 at centromere/kinetochores in human cells. (A) HeLa cells treated with DMSO or ZM447439 immunostained for Histone H3 P-Ser10 (green), Plk1 (red), or Plk1T210Ph (red) and DNA (blue). Scale bar = 10 µm. (B) Quantification graph of Plk1 and Plk1T210Ph levels at centromeres in Control and ZM447439 treated cells. Fluorescent intensities are in Arbitrary Units (A.U.). t test: *** p<0.0001. (TIF) [file pbio.1001250.s008.tif]
