# Supplementary figures and images for: Correction: The Chromosomal Passenger Complex Activates Polo Kinase at Centromeres
Source: PLoS Biol. 2012 Feb 14;10(2):10.1371/annotation/7832f573-e0d9-465f-b5d4-0ac1014b6112. doi: 10.1371/annotation/7832f573-e0d9-465f-b5d4-0ac1014b6112 (PMC3284318; doi:10.1371/annotation/7832f573-e0d9-465f-b5d4-0ac1014b6112)

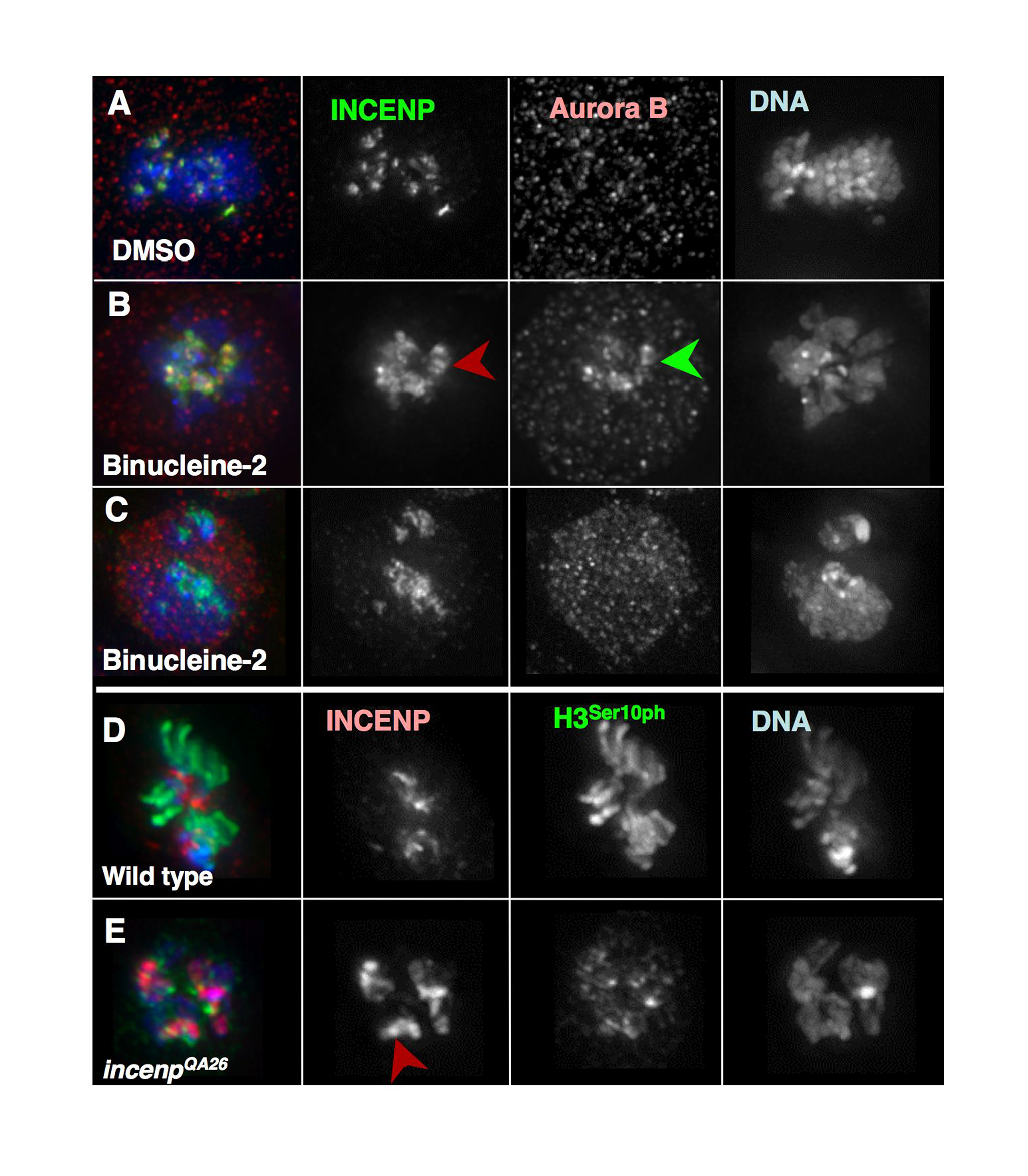

Supplement: Supplementary file 1 [file pbio.7832f573-e0d9-465f-b5d4-0ac1014b6112.s001.tif]
